# Analysis of Poly(ADP-Ribose) Polymerases in *Arabidopsis* Telomere Biology

**DOI:** 10.1371/journal.pone.0088872

**Published:** 2014-02-13

**Authors:** Kara A. Boltz, Madhu Jasti, Jennifer M. Townley, Dorothy E. Shippen

**Affiliations:** Department of Biochemistry and Biophysics, Texas A&M University, College Station, Texas, United States of America; Tulane University Health Sciences Center, United States of America

## Abstract

Maintaining the length of the telomere tract at chromosome ends is a complex process vital to normal cell division. Telomere length is controlled through the action of telomerase as well as a cadre of telomere-associated proteins that facilitate replication of the chromosome end and protect it from eliciting a DNA damage response. In vertebrates, multiple poly(ADP-ribose) polymerases (PARPs) have been implicated in the regulation of telomere length, telomerase activity and chromosome end protection. Here we investigate the role of PARPs in plant telomere biology. We analyzed *Arabidopsis thaliana* mutants null for *PARP1* and *PARP2* as well as plants treated with the PARP competitive inhibitor 3-AB. Plants deficient in PARP were hypersensitive to genotoxic stress, and expression of *PARP1* and *PARP2* mRNA was elevated in response to MMS or zeocin treatment or by the loss of telomerase. Additionally, *PARP1* mRNA was induced in *parp2* mutants, and conversely, *PARP2* mRNA was induced in *parp1* mutants. *PARP3* mRNA, by contrast, was elevated in both *parp1* and *parp2* mutants, but not in seedlings treated with 3-AB or zeocin. PARP mutants and 3-AB treated plants displayed robust telomerase activity, no significant changes in telomere length, and no end-to-end chromosome fusions. Although there remains a possibility that PARPs play a role in Arabidopsis telomere biology, these findings argue that the contribution is a minor one.

## Introduction

The essential functions of telomeres are to promote complete replication of the chromosome terminus and to distinguish the natural ends of chromosomes from DNA double-strand breaks (DSBs). Telomeres consist of simple G-rich repeat DNA that is synthesized and maintained by the telomerase reverse transcriptase. Telomerase docks on the 3′ single-strand (ss) extension on the chromosome end (G-overhang) via contacts with telomere binding proteins. The two main telomere protein complexes are shelterin and CST. Vertebrate shelterin is composed of six core subunits including the double-strand (ds) DNA binding TRF1 and TRF2 (reviewed in [1]). Although the CST (CTC1/STN1/TEN1) complex, which associates with the G-overhang, was first identified in budding yeast, CST-related components have now been identified in *Schizosaccharomyces pombe*, vertebrates, and plants [2]–[6]. *Arabidopsis thaliana* encodes at least six TRF-like proteins [7], [8], but CST seems to be the primary factor required for telomere integrity. Loss of any of the three CST proteins in plants leads to dramatic telomere shortening, end-to-end chromosome fusions and severe developmental defects that culminate in stem cell failure [3]–[5]. In vertebrates, shelterin plays a more significant role in promoting telomere stability than CST, which acts primarily to facilitate telomeric DNA replication [9]–[11]. Thus, while core components of the telomere complex are conserved, their specific contributions to telomere biology are evolving.

Curiously, although a major function of telomeres is to distinguish chromosome ends from DNA damage [12], [13], multiple DNA repair-related proteins are vital for normal telomere function. The phosphoinositide-3-kinase-related protein kinase ATM (Tel1 in yeast) responds to DSBs, and yet is required for telomerase action at chromosome ends [14]–[16]. Likewise, the related kinase ATR, which is activated by ssDNA breaks (SSB), is implicated in telomerase recruitment [16], [17] as well as promoting DNA replication through the ds portion of the telomere [18]–[20]. The Ku70/80 heterodimer is required for the classic non-homologous end joining (NHEJ) pathway of DSB repair, but also has multiple functions at telomeres. Ku protects chromosome ends, particularly the extreme 5′ terminus [21], [22]. Ku also interacts with the telomerase RNA subunit [23]–[25] and recruits telomerase to budding yeast telomeres in the G1 phase of the cell cycle [26].

Another group of repair-related proteins required for telomere function in human cells are the poly(ADP-ribose) polymerases (PARPs) [27]. PARPs are found in all eukaryotic supergroups [28] and catalyze the synthesis and transfer of poly ADP-ribose (PAR) from NAD^+^ to target proteins. PARylation can alter the function of proteins in several ways [29]. It adds a negative charge to proteins and can cause a protein to dissociate from its binding partner, often DNA, and can also promote protein complex formation through recruitment of PAR binding proteins [30], [31]. PARylation can also mark proteins for destruction by recruiting PAR-binding E3 ubiquitin ligases [32], [33].

Although they are best known for their role in DNA repair, particularly Base Excision Repair and other types of ssDNA repair, PARPs are reported to function in other cellular processes, such as mitosis, regulating chromatin state, and transcription [34]–[39]. In addition, PARPs are important for cellular responses to many types of environmental assaults, including heat, genotoxic, metabolic, and oxidative stresses [40]–[43]. Low PARP activity promotes cellular survival through activation of pathways to resolve cellular stress, but high levels of PARP activity, which results in high levels of PARs, are an indication that stress levels have overwhelmed the cell. PARPs can activate cell death in at least two ways. Because PARPs use NAD^+^ as a substrate, high levels of PARP activity can rapidly deplete ATP, causing an energy crisis which leads to necrotic cell death [40], [44]. Additionally, overabundance of free PARs induces caspase-independent cell death [40], [44].

Several of the mammalian PARPs have been implicated in telomere length regulation, chromosome end protection, and telomerase regulation [45], [46]. PARP proteins mediate their telomere functions primarily via interactions with the shelterin components TRF1 and TRF2. The first identified telomere-associated PARP, Tankyrase1 (TRF1-interacting, ankyrin-related ADP-ribose polymerase), was discovered as an interaction partner of TRF1 [47]. Human TRF1 is both a binding partner and a PARylation target of Tankyrase1 [47], [48]. PARylation of TRF1 leads to its dissociation from telomeres [47], [48] and its subsequent ubiquitination and proteolytic destruction [49]. Loss of TRF1, a negative regulator of telomere length, causes telomere elongation, presumably by increasing telomerase access to the telomeres [50], [51]. Additionally, PARylation of TRF1 by Tankyrase1 is required for resolving sister telomere cohesion during mitosis [52], [53]. The closely related protein Tankyrase2 shows similar localization and function as Tankyrase1 in human cells [51], [54].

Three other members of the PARP superfamily in humans, PARP1, PARP2, and PARP3, have also been studied in the context of telomere biology. PARP1 and PARP2 bind to TRF2 and PARylate it *in vitro* [55], [56]. Similarly to TRF1, PARylation of TRF2 causes it to dissociate from telomeric DNA [55], [56]. PARP1 has been proposed to modulate telomerase activity [57], [58]. It associates with the telomerase catalytic subunit TERT (Telomerase Reverse Transcriptase) *in vivo* [59], and binds directly to a TERT peptide *in vitro* [60]. PARP1 may also have an important role at damaged telomeres. In human cells, telomere-association of PARP1 increases after treatment with DNA damaging agents. PARP1 association with telomeres is also enhanced in mouse cells with critically shortened telomeres due to telomerase inactivation [56] [56]. PARP3 is the newest PARP shown to affect human telomere function. Knockdown of *PARP3* results in sister telomere fusions and telomere loss in mitotic spreads [61]. PARP3 interacts with Tankyrase1 and is thought to function at telomeres by stimulating activation of Tankyrase1 [61].

Many components of the mammalian DDR are conserved in plants, but less is known about the details of the plant DDR. One remarkable feature that distinguishes plants from animals is their high tolerance to genome instability. This tolerance may arise from the maintenance of undifferentiated stem cell niches throughout the plant life cycle. Accordingly, DNA damage in vegetative organs may not have a major impact on survival because plants can compensate by initiating new growth and tissue differentiation. In gamma radiation-treated plants, for example, cell cycle arrest is induced in meristems, but not in somatic cells [62]. In addition, programmed cell death (PCD) is initiated in response to DNA damage via ATM and ATR, which also contributes to genome preservation in plant stem cells by culling out cells with unrepaired DNA damage [63]–[66].

*Arabidopsis thaliana* has proven to be an excellent model system for telomere analysis because of its high tolerance to genome instability and telomere dysfunction. Unlike budding yeast [67], [68], *Arabidopsis* mutants lacking core components of CST are viable and semi-fertile for a few generations even though they suffer severe telomere dysfunction [3], [4]. Further, plants can survive without key DNA damage response proteins. *Arabidopsis* lacking ATM and ATR are viable under normal growth conditions, although *atm* mutants have reduced fertility [69], [70]. In striking contrast, loss of ATR is lethal in vertebrates [71]. *Arabidopsis* is thus a good choice for comparative studies of the telomere-related function of PARPs in a divergent multicellular eukaryote.

The PARP gene family is considerably smaller in plants than in vertebrates. *A. thaliana* encodes nine PARP proteins and strikingly none of these bear the signature of tankyrase-like PARPs. *Arabidopsis* also lacks a homolog to human PARP2. Three of the *Arabidopsis* PARPs (AtPARP1, AtPARP2, AtPARP3) have confirmed or predicted poly ADP-ribosylation activity, whereas the other six are predicted to lack enzymatic activity [72]. Of the three PARPs with enzymatic activity, AtPARP2 is homologous to HsPARP1, while AtPARP1 and AtPARP3 more closely resemble HsPARP3. Both AtPARP1 and AtPARP2 are ubiquitously expressed, but AtPARP3 expression is confined to seeds under standard growth conditions [73].

Plant PARPs have been studied mostly in the context of biotic and abiotic stress [74]–[77]. As mentioned above for vertebrates, plant PARPs are stimulated by multiple types of stress and can promote either cell survival or cell death. AtPARP1 and AtPARP2 localize to the mitotic spindle and thus may have functions similar to those of Tankyrase1 and human PARP3 in preventing fusion of sister chromatids during cell division [73]. Indirect evidence indicates that AtPARP1 and AtPARP2 function in DNA repair *in vivo*. Both PARPs are highly expressed after induced DNA damage and replication stress, and AtPARP2 binds to DNA breaks [70], [73], [78]. Recently, AtPARP1 and AtPARP2 were shown to play a role in microhomology-mediated end joining *in vitro* [79].

Here we analyze the three enzymatically-active PARPs to examine the role of PARPs in *Arabidopsis* telomere biology. In seedlings, induction of DNA damage with MMS or zeocin causes increased expression of *PARP1* and *PARP2*, but not *PARP3*. Further analysis of PARP expression revealed that absence of PARP1 or PARP2 leads to increased expression of the other two PARPs. We further show that in plants carrying a null mutation in TERT, PARP transcripts are upregulated, indicating that telomere dysfunction can also trigger PARP activation. Finally, using PARP mutants as well as PARP-inhibitor treated seedlings, we demonstrate that PARPs make no significant contribution to regulating telomerase enzyme activity, controlling telomere length or protecting chromosome ends from end-joining reactions. We conclude that the role of PARPs in modulating the DDR is conserved, but their telomere-related functions are not.

## Materials and Methods

### Plant Materials and Growth Conditions

T-DNA lines for AtPARP1 (SALK_ 140400) and for AtPARP2 (GABI_380E06-017222) were obtained from the *Arabidopsis* Biological Resource Center (ABRC). The SALK line was recently characterized by another group [79]. The *tert* mutant [80] and *ku70* mutant [22] and their phenotypes were described previously. Double *parp1 parp2* mutants were made by crossing a homozygous *parp1* mutant with a homozygous *parp2* mutant. Double heterozygous F1 plants were identified by genotyping and then self-propagated to F2 to obtain double homozygous mutants. Plants were grown at 23°C in an environmental chamber under a 16 h light/8 h dark photoperiod.

The primers for genotyping were: PARP1: 5′-TTTTCTCTTCGTTCAAATGTGC-3′ and 5′-TGTAGCGACAAACCATTGCT-3′ in conjunction with Salk T-DNA primers Lba1 and Lbb1.3. PARP2: 5′-ACTCCTCAAGGAGTGAAAGGC-3′ and 5′-ACTCCATCATTGCAGATCTGC-3′ in conjunction with GabiKat T-DNA primers 3144 and 8409.

### Chemical Treatments

Seeds were sterilized and germinated on solid 0.5X MS (Murashige and Scoog) medium. Five days after germination, seedlings were transferred to liquid MS medium containing 0, 25, 50, 75 or 100 ppm MMS (Sigma). Seedlings were collected and analyzed or frozen immediately after the MMS treatment. Seedlings were treated with MMS for five days for measurement of DNA damage response and for one week to score MMS sensitivity.

For 3-AB treatment, seeds were sown directly into liquid 0.5X MS plus 5 mM 3-AB (Sigma)/0.6% DMSO or MS plus 0.6% DMSO and were grown for one week under constant light with gentle shaking. To induce DNA damage in 3-AB treated and control seedlings, 20 µM zeocin (Invitrogen) was added for four hours before harvesting the seedlings.

### RNA Extraction and RT-PCR Analysis

Frozen seedlings were finely ground and RNA was extracted using TRI reagent (Sigma). The extracts were treated with RQ1 DNAse (Promega) for 1 hr. cDNA was synthesized using Superscript III reverse transcriptase (Invitrogen) with oligo (dT) primer. DNA damage responses were measured in MMS treated wild type and *tert* seedlings by checking the mRNA levels of *PARP2*. The reaction mixture was amplified with Taq polymerase for 20 cycles of PCR at 94°C for 3 min, 55°C for 40 sec and 72°C for 1 min 15 sec with a final extension time at 72°C for 5 min. The entire reaction was resolved on a 1% agarose gel and subjected to Southern blot with a *PARP2* cDNA probe labeled with [α-^32^P]-dCTP. As a loading control, RT-PCR was performed with primers specific for *Actin-2*.

For all other RT-PCR experiments, RNA was extracted with the Direct-Zol RNA Miniprep Kit with on-column DNAse treatment (Zymo Research). 1 µg of total RNA was used with the qScript cDNA Supermix (Quanta Biosciences). The resulting cDNA was diluted 1∶4 in 10 µg/ml yeast tRNA and 4 µL was used for qPCR. qPCR was run on a CFX Connect Real-Time PCR Detection System (Bio-Rad) using SsoAdvanced SYBR Green Supermix (Bio-Rad) following the manufacturer’s suggested protocol. Each reaction was run in duplicate and later averaged, and at least three biological replicates were run for each experiment. A reference gene that is reported to have steady levels of transcription in many conditions [81], *PDF2*, was run for each sample. LinRegPCR was used with default settings to calculate initial transcript levels (N_0_) that were corrected for PCR efficiency. To correct for loading the target N_0_ value was divided by the reference gene N_0_ value. This value was then divided by the average corrected value for the control sample (wild type or untreated).

qRT-PCR primers were: PARP1: 5′- ATGCTACTCTGGCACGGTTCAC-3′ and 5′- AGGAGGAGCTATTCGCAGACCTTG-3′. PARP2: 5′- ATCGTCTACGATACAGCCCAGGTG-3′ and 5′-TGGTTCAGGCTCATCTCTTGTGC-3′. PARP3: 5′- CGTCAAAGATGGTGGAGGCAATGG-3′ and 5′-TCGATCAACCATGCTTCGCTCAC-3′. BRCA1: 5′-TGCATCCATTAAGTTGCCCTGTG-3′ and 5′- TAGGCTGAGAGTGCAGTGGTTC-3′. ACT2: 5′-CTTGCACCAAGCAGCATGAA-3′ and 5′-CCGATCCAGACACTGTACTTCCTT-3′. PDF2: 5′-TAACGTGGCCAAAATGATGC-3′ and 5′-GTTCTCCACAACCGCTTGGT-3′.

### TRAP (Telomere Repeat Amplification Protocol)

Protein was extracted from flowers or seedlings using Buffer W as previously described. qTRAP was performed as previously described [82]. For radioactive TRAP extracts were diluted 1∶10, and for quantitative TRAP (qTRAP) 50 ng of total protein was used for each sample. The extract, telomere oligo substrate, and α-[^32^P]dGTP were added to Hot Start GoTaq master mix (Promega) and incubated for 45 minutes at 37°C. TRAP reverse primer was then added to each reaction and then PCR was run. Products were precipitated with ethanol/sodium acetate (pH 5.2)/glycogen and run on a 6% polyacrylamide, 7M urea sequencing gel.

### Telomere Length Measurement and Telomere Fusion PCR

Genomic DNA was extracted from seedlings or whole plants using 2x CTAB buffer [83]. TF-PCR and PETRA [84] and TRF [85] were conducted as previously reported. For all three assays, products were detected by Southern blot with a [^32^P]-5′-end-labeled (TTTAGGG)_4_ probe.

## Results

### Generation of Plants Null for PARP Activity

**Figure 1:**
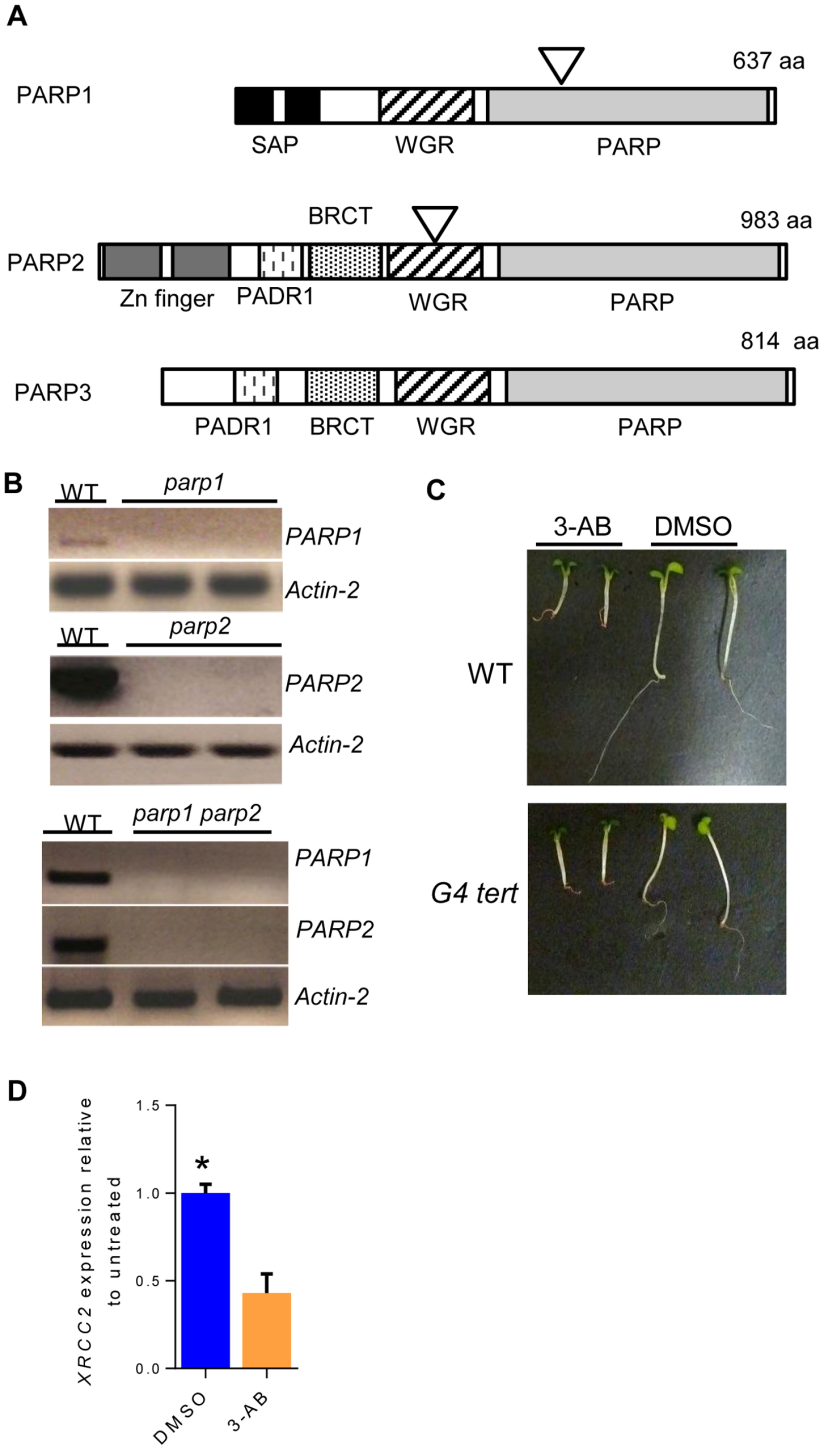
Characterization of T-DNA mutants and 3-AB treated seedlings. (A) Schematic of PARP proteins. The triangles indicate the position of the T-DNA insertions. The T-DNA for PARP1 is located in the intron between exons 6 and 7, within the PARP catalytic domain. The T-DNA for PARP2 is located in exon 10, within the WGR domain. SAP: SAF-A/B, Acinus and PIAS (nucleic acid-binding domain); WGR: Named after conserved central motif (Trp, Gly, Arg) (putative DNA-binding domain); PARP: PARP regulatory and catalytic domain; PADR1: Domain of unknown function found in PARPs; BRCT: BRCA1 C-terminus. (B) Semi-quantitative RT-PCR for *PARP1* and *PARP2* expression levels in *parp1* (top), *parp2* (middle) and *parp1 parp2* double mutants (bottom). *Actin-2* served as a loading control. (C) Wild type (top) and G4 *tert* mutant seedlings (bottom) grown in 5 mM 3-AB/0.6% DMSO (left) or in 0.6% DMSO (right). (D) qRT-PCR for *XRCC2* expression in 3-AB-treated wild type seedlings relative to untreated seedlings. * = p-value <0.05 by Student’s two-tailed t-test.

To examine the role of PARP proteins at *Arabidopsis* telomeres, we sought to identify mutants lacking *PARP1* or *PARP2*. T-DNA insertion lines were obtained for both *PARP1* (At4G02390) and *PARP2* (At2G31320) (Figure 1A). *PARP1* and *PARP2* transcription was abolished in single *parp1-1* and *parp2-1* mutants as indicated by RT-PCR analysis (Figure 1B). To investigate the combined contribution of PARP1 and PARP2, we generated a *parp1 parp2* double mutant by genetic crossing. RT-PCR analysis confirmed that expression of both *PARP1* and *PARP2* was abolished in the double mutants (Figure 1B).

During the course of this study, PARP3 was identified in vertebrates [61]. A putative ortholog, At5g22470, is also present in *A. thaliana*. Because of the difficulties in generating triple mutants, we instead chose to use the PARP inhibitor 3-AB (3-aminobenzamide) on wild type plants to eliminate PARP enzymatic activity. 3-AB, a competitive inhibitor of PARP enzymatic activity which prevents PARP binding to NAD^+^, has been previously employed in plant studies [76], [86] and was also used in several studies of the telomeric function of PARP in mammalian cell culture [57]. Seeds were sown in liquid MS with either 3-AB (in DMSO) or DMSO only. Seedlings were collected seven days later. In contrast to previous reports of enhanced growth with the PARP-inhibitor 3-MB (3-methoxy-benzamide) [87], our 3-AB treated seedlings had diminished root growth compared to untreated seedlings (Figure 1C). Shoots were also smaller in the 3-AB-treated seedlings, but this could reflect a defect in nutrient uptake caused by the small roots. To further verify the action of 3-AB, we used qRT-PCR to measure levels of the *XRCC2* mRNA, a DNA repair gene, which is reported to be downregulated in response to 3-AB treatment in *Arabidopsis* [86]. As expected, our 3-AB treated samples showed a decrease in the levels of *XRCC2* transcripts similar to previous reports (Figure 1D) [86]. Although we cannot rule out the possibility that residual PARP activity remains, the transcriptional analysis (also see below) of 3-AB treated plants indicate that the PARP inhibitor worked as expected.

### PARP Mutants are Sensitive to Genotoxic Stress

**Figure 2:**
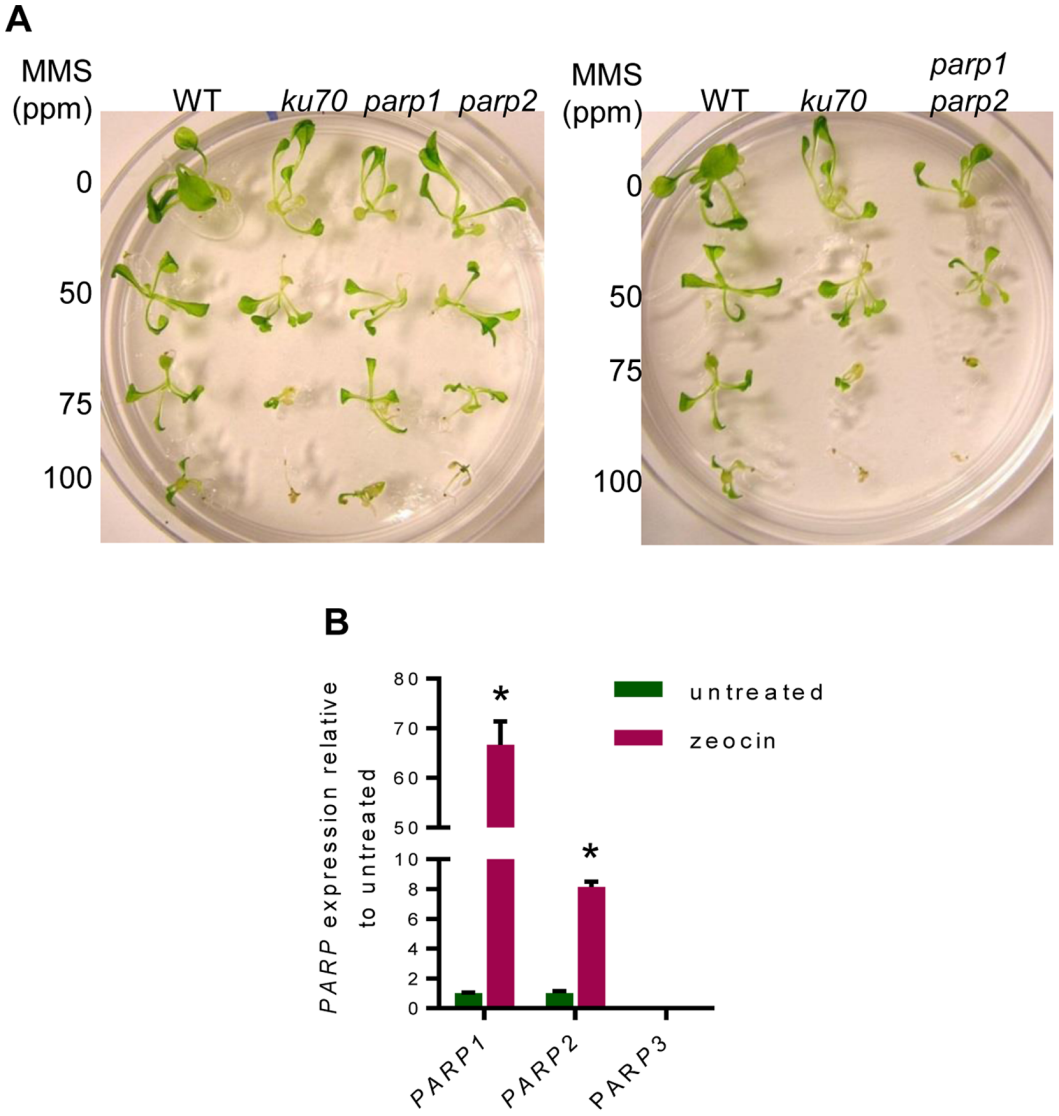
*Arabidopsis* PARPs respond to genotoxic stress. (A) Morphological and developmental defects of seedlings grown in increasing concentrations of MMS. Left panel: *parp1* and *parp2* single mutants. Right panel: *parp1 parp2* double mutants. *ku70* mutants were used as a positive control for MMS sensitivity. (B) qRT-PCR for *PARP* expression in wild type seedlings. Seedlings were either treated with 20 µM zeocin/0.6% DMSO or 0.6% DMSO (untreated) for 4 h. *PARP3* expression was not detected. * = p-value <0.005 compared to untreated.

Because PARP proteins are important for the response to ssDNA damage, we verified that our mutants were sensitive to genotoxic stress by treating five-day-old seedlings with increasing concentrations of the DNA alkylating agent methyl methane sulfonate (MMS). Growth and morphology of *parp1* and *parp2* mutants were compared to wild type and *ku70* seedlings, which are hypersensitive to MMS [22]. At all three MMS concentrations tested, *parp1* and *parp2* mutants were smaller and less developed than wild type seedlings, but were not affected as much as the *ku70* mutants (Figure 2A). Notably, the *parp1 parp2* double mutants were more sensitive than either single mutant. The double mutants were similarly or slightly more sensitive to MMS than the *ku70* mutants (Figure 2A). During the preparation of this manuscript, Jia *et al* [79] published a study which included measurements of MMS-sensitivity in *A. thaliana* PARP mutants. These authors found that the single *parp1* or *parp2* mutants did not differ significantly from wild type, but double *parp1 parp2* mutants had reduced root growth [79]. Our disparate results may arise from analysis of different mutant alleles as well as procedural variation, including the amount and duration of MMS treatment. Nevertheless, the differences between the two studies do not change the overall conclusion that *parp1* and *parp2* mutants are sensitive to DNA damage, and this sensitivity increases when both PARPs are lost.

### Expression of *PARP1* and *PARP2*, but not *PARP3*, is Induced by Genotoxic Stress

To further explore the response of PARPs to DNA damage in *Arabidopsis*, seven-day-old wild type seedlings were grown in liquid MS containing zeocin, a radiomimetic drug that induces DSBs. Expression levels for *PARP1*, *PARP2*, and *PARP3* were then measured by qRT-PCR. Compared to untreated controls, *PARP1* expression was increased 66-fold (p-value = 0.00015) and *PARP2* increased by 8-fold (p-value = 0.0001) (Figure 2B). Although the increase in *PARP1* and *PARP2* was expected based on previous reports [88], [89], the expression of *PARP3* in response to DSBs has not been described. In either treated or untreated seedlings, the qRT-PCR signal for *PARP3* was either not detected or was extremely low (C_t_ ≥34) (Figure 2B), suggesting that PARP3 is not upregulated in response to DNA damage in seedlings, and therefore has separate functions from PARP1 and PARP2.

### *Arabidopsis* PARPs Negatively Regulate Expression of Each Other

**Figure 3:**
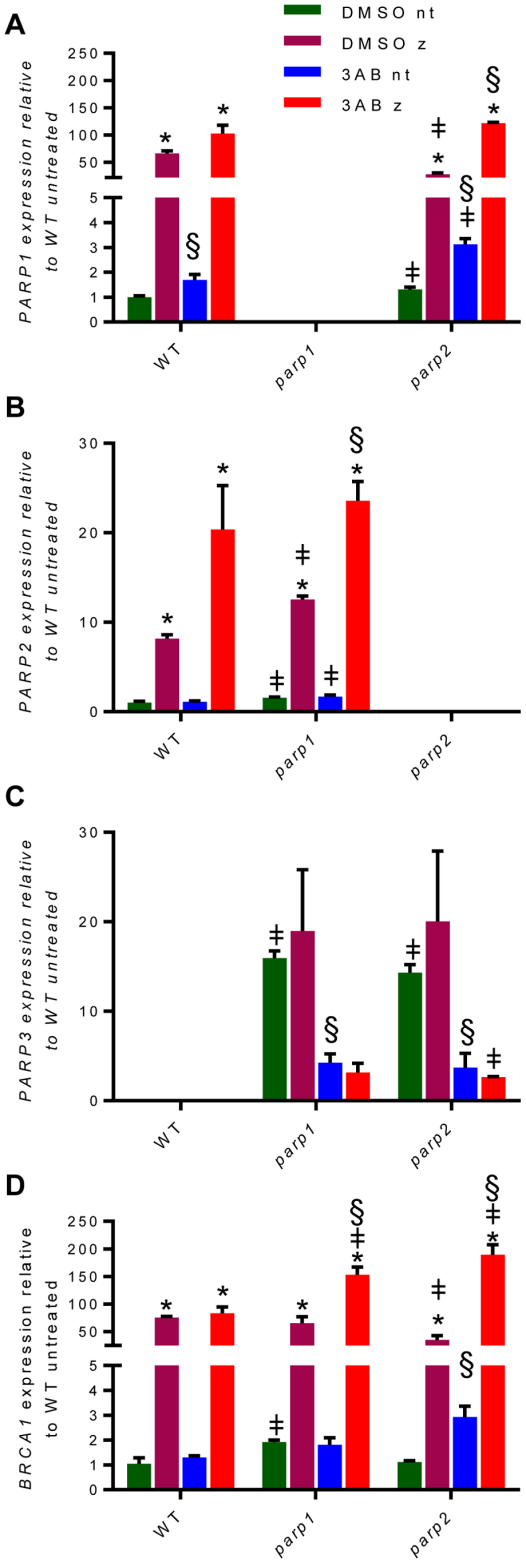
*PARP1* and *PARP2* negatively regulate expression of each other and *PARP3*. qRT-PCR results for *PARP1* (A), *PARP2* (B), *PARP3* (C), and *BRCA1* (D) transcripts in seedlings treated with 3-AB and zeocin. All values were calculated relative to wild type seedlings that were not treated with either 3-AB or zeocin. DMSO = no 3-AB; nt = not treated with zeocin; z = zeocin treatment. Student’s two-tailed t-tests were used for statistical analysis. P-values <0.05 are indicated by symbols above the bars for the following comparisons: ‡ = mutant compared to wild type with the same treatment; * = untreated compared to zeocin treated for the same genotype and 3-AB status; § = DMSO compared to 3-AB treated for the same genotype and zeocin status.

Because our experiments with MMS and zeocin showed similar phenotypes for plants lacking PARP1 and PARP2, we wondered whether either of the two PARPs might compensate for the loss of the other through increased expression. qRT-PCR was used to measure mRNA levels of all three PARPs in wild type, *parp1, parp2*, and 3-AB treated seedlings (Figure 3). In untreated seedlings, *PARP1* expression in *parp2* mutants was slightly higher (1.5-fold, p-value = 0.03) than in wild type plants (Figure 3A). *PARP2* expression in *parp1* mutants was similarly increased (1.6-fold, p-value = 0.02) (Figure 3B). Although the increased expression is small, it is consistent with the hypothesis that PARP1 and PARP2 negatively regulate the expression of each other.

*PARP3* is normally only expressed in *Arabidopsis* seeds [73], and was not detectable in wild type seedlings treated with zeocin (Figure 2B). To assess whether *PARP3* expression changes in the absence of PARP1 or PARP2, we monitored *PARP3* expression in *parp1* and *parp2* mutants. In *parp1* and *parp2* seedlings, the levels of *PARP3* transcript increased dramatically (Figure 3C). Although the increase appeared to be 14 to 16-fold compared to wild type (Figure 3C), the qRT-PCR signal was barely detected in wild type, and thus it is possible that the induction of *PARP3* is even higher. We can conclude, however, that the absence of PARP1 or PARP2 leads to an induction of *PARP3* expression in seedlings. Thus, PARP3 may have some similar functions with PARP1 and PARP2 that are dormant in wild type seedlings.

Taken together, our transcriptional analysis suggests that the PARPs negatively regulate each other. How this regulation is achieved is unknown. One possibility is that transcription of the *PARP* genes is controlled by a feedback loop in which ADP-ribosylation of some unknown factor influences *PARP* expression. Alternatively, PARPs could have non-catalytic functions such as binding promoters that influence gene expression. Finally, the increase in *PARP* expression could reflect an increase in background levels of DNA damage caused by the absence of one or more *PARPs*, with the increased DNA damage, in turn, causing upregulation of other PARPs. To differentiate between these possibilities, we measured mRNA levels in wild type, *parp1* and *parp2* seedlings that were grown +/−3-AB and +/− zeocin.

The level of DNA damage after zeocin treatment was monitored by measuring the induction of the DNA repair gene *BRCA1* (Figure 3D). In the absence of zeocin in *parp1* mutants, *BRCA1* was slightly increased (1.9-fold, p-value = 0.0009) relative to wild type (Figure 3D, blue and green bars), suggesting that the absence of PARP1 is associated with increased background DNA damage levels. This finding is consistent with a recent report of higher DNA damage in *A. thaliana parp* mutants as measured by the comet assay [79]. When mutant seedlings were treated with 3-AB to inhibit PARP activity as well as with zeocin to induce DNA damage, *BRCA1* transcript levels were increased compared to zeocin alone (Figure 3D, red and magenta bars). Altogether these results support the conclusion that PARP1 and PARP2 limit the amount of DNA damage in cells.

The effect of 3-AB treatment on *PARP* expression levels varied between the three genes. In the absence of DNA damage, *PARP1* expression increased compared to untreated controls in both wild type and *parp2* mutants (1.7 and 3.1-fold, respectively), whereas *PARP2* levels were similar with or without 3-AB (Figure 3A and B, blue and green bars). *PARP3* levels, conversely, were dramatically decreased compared to non-3-AB treated controls (Figure 3C), suggesting that PARP activity is needed for the increase in *PARP3* expression seen in *parp1* and *parp2* mutants.

In seedlings treated with both zeocin and 3-AB, *PARP1* and *PARP2* expression increased more than when only zeocin was present (Figure 3A–B). This outcome could result from higher levels of DNA damage due to PARP inhibition. As seen with wild type (Figure 2B), zeocin did not induce additional *PARP3* expression in *parp1* or *parp2* mutants (Figure 3C). We conclude that in this developmental stage, PARP3 is not responsive to DNA damage.

### PARPs are Upregulated in Response to Telomerase Inactivation

**Figure 4:**
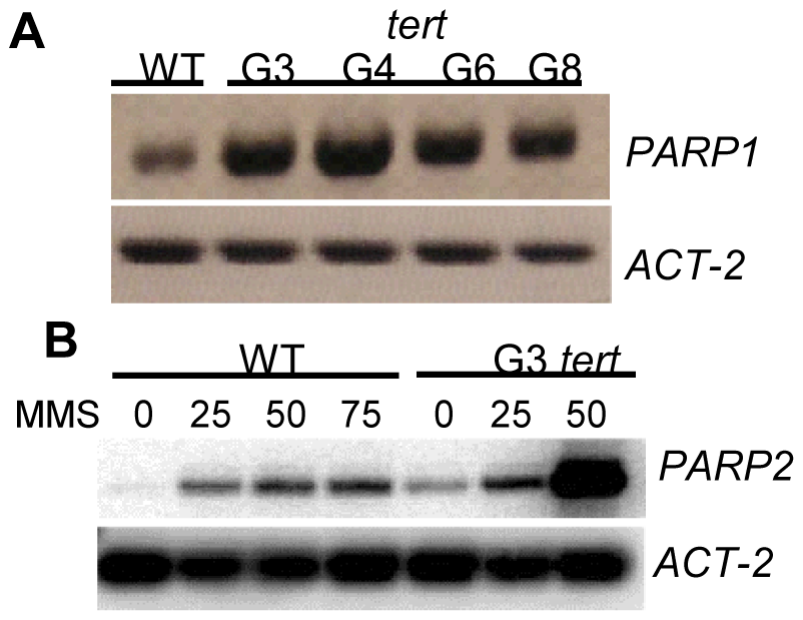
*Arabidopsis* PARPs respond to the absence of telomerase. (A) RT-PCR of *PARP1* transcript levels in different generations of *tert* mutants. (B) RT-PCR of *PARP2* expression in wild type and 3^rd^ generation (G3) *tert* mutants at increasing concentrations of MMS.

We previously reported that *PARP1* mRNA is induced in response to telomere dysfunction triggered by prolonged telomerase inactivation [23] or by loss of a core component of the CST telomere capping complex [64]. To further explore the PARP1 response, we used RT-PCR to examine *PARP1* expression in four different generations of *tert* mutants: generation 3 (G3), G4, G6 and G8. Although the onset of telomere dysfunction is somewhat stochastic, early generation telomerase-deficient plants have shorter telomeres that are refractory to chromosome end-joining reactions, while the extremely short telomeres in later generation mutants cause chromosome end de-protection and massive genome instability [80]. Notably, *PARP1* levels were elevated in all generations of the mutants tested (Figure 4A). *PARP2* was also upregulated in G3 *tert* mutants compared to wild type seedlings (Figure 4B), indicating that the loss of telomerase leads to a DDR where both *PARP1* and *PARP2* are upregulated. The induction of *PARP2* was even higher in G3 *tert* mutants treated with MMS (Figure 4B). At 50 ppm of MMS, G3 *tert* mutants showed a much larger increase in *PARP2* levels compared to wild type (Figure 4B). Thus, the absence of telomerase renders plants hypersensitive to DNA damage and triggers a dramatic upregulation of *PARPs*.

### PARPs are not Required for Telomerase Activity in *Arabidopsis*

**Figure 5:**
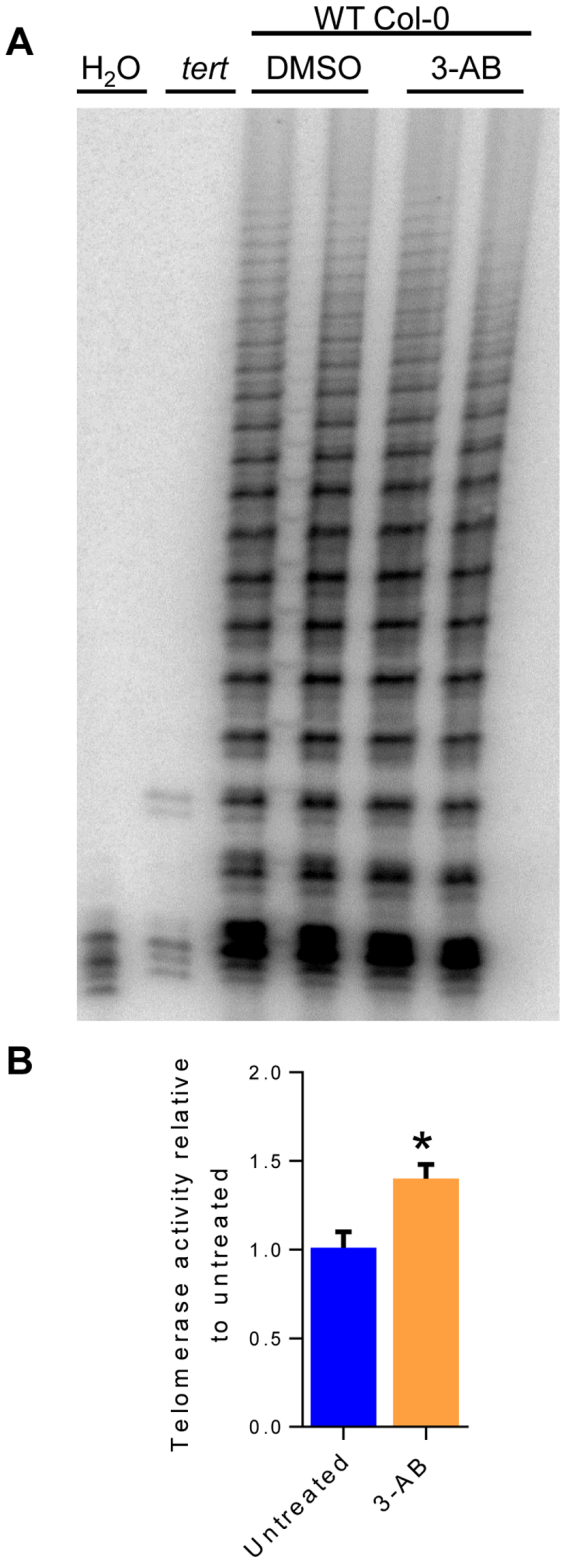
*Arabidopsis* telomerase is not stimulated by PARPs. (A) TRAP analysis on seedlings. Seedlings were treated with either 3-AB (WT) or DMSO (WT and G4 *tert* mutants) (B) Quantitative TRAP results for 7-day-old 3-AB-treated wild type seedlings relative to untreated seedlings. P-value = 0.03 by Student’s two-tailed t-test.

Some reports with human cells indicate that PARPs stimulate telomerase activity [58], [90]. Therefore, we monitored telomerase activity in plants deficient in PARP using the Telomeric Repeat Amplification Protocol (TRAP). Telomerase activity was detected in seedlings treated with 3-AB (Figure 5A) as well as in plants doubly deficient for *parp1* and *parp2* (data not shown). Quantitative TRAP (qTRAP) performed on seedlings treated with 3-AB revealed a slight increase in telomerase activity (1.4-fold, p-value = 0.03) relative to untreated seedlings (Figure 5B). These data are in accordance with the majority of studies in mammalian systems which found no change in telomerase activity levels when PARPs were inhibited or mutated [55], [57], [91]–[93].

### PARPs do not Make a Significant Contribution to Telomere End Protection in *Arabidopsis*

**Figure 6:**
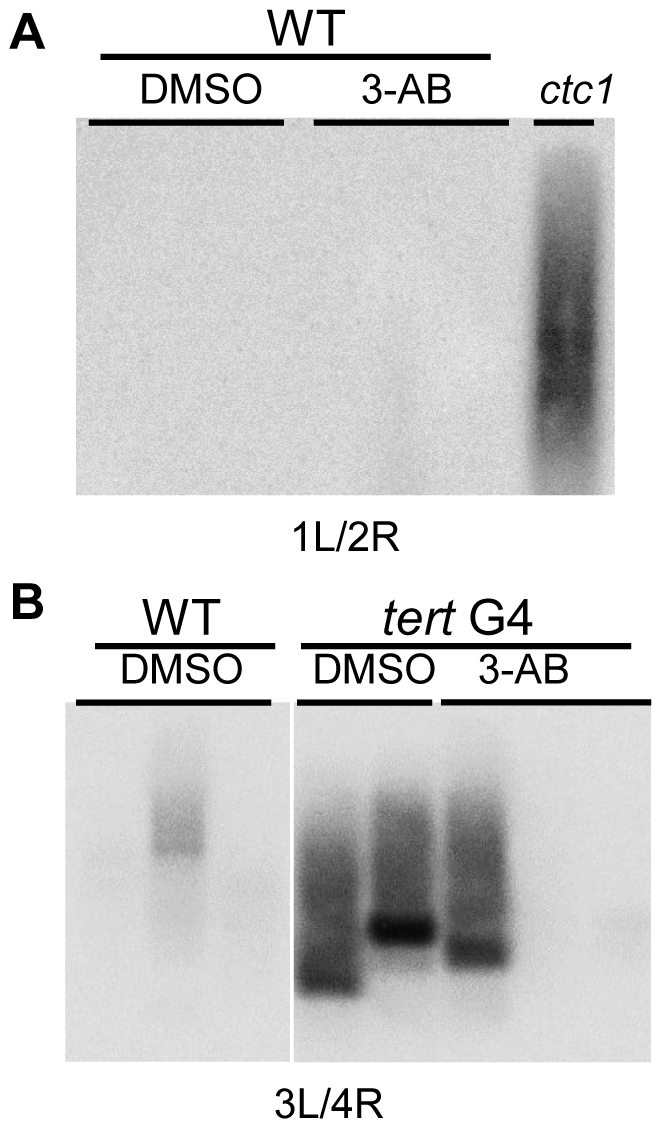
PARPs are not required to prevent end-to-end chromosome fusions. Telomere fusion PCR for (A) 3-AB treated wild type seedlings, and (B) 3-AB treated *tert* mutants. The pairs of subtelomeric primers used for TF- PCR are indicated below each blot, where 1L refers to the left arm of chromosome 1 and 2R to the right arm of chromosome 2, etc. *ctc1* mutants were used as a positive control.

We next asked whether PARPs contribute to chromosome end-protection and genome stability in *Arabidopsis*. We performed cytogenetic analysis on dissected pistils from *parp1* and *parp2* single and double mutants. Anaphase bridges are the hallmark of dysfunctional telomeres, reflecting the formation of dicentric chromosomes that arise from the fusion of deprotected chromosome ends. We found no mitotic abnormalities, including anaphase bridges, in either *parp1* or *parp2* single mutants or the double mutant (data not shown). A more sensitive assay to detect end-to-end chromosome fusions is telomere fusion PCR (TF-PCR) [84]. If unprotected or short telomeres are covalently joined together, the chromosome junction can be amplified using primers specific to subtelomeric sequences on individual chromosome arms. In the absence of end joining, no PCR product will be generated. In contrast to the control reaction performed with a mutant lacking the CST component CTC1, TF-PCR failed to reveal evidence for telomere fusions in either the *parp1* or *parp2* mutants (data not shown) or seedlings treated with 3-AB (Figure 6A). These results are consistent with our cytological analysis and indicate that telomere protection is intact in plants lacking PARPs. Alternatively, if PARPs promote telomere protection in a subset of cells, such as in the root meristem, the phenotype could have been diluted out in our assays, which used whole seedlings or flowers.

**Figure 7:**
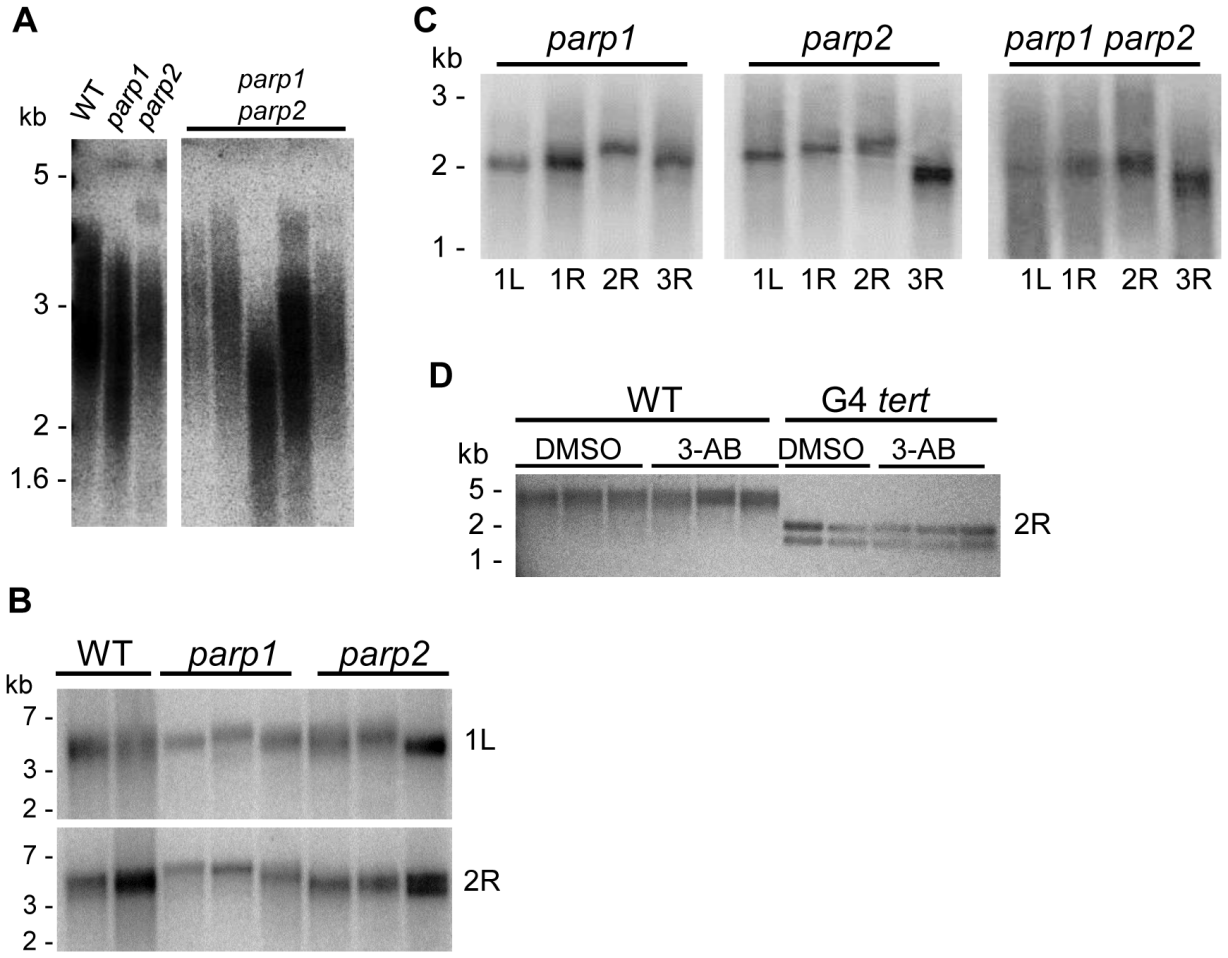
PARPs are not required to maintain telomere length in *Arabidopsis*. (A) TRF analysis of bulk telomeres in several individual wild type, *parp1*, *parp2,* and *parp1 parp2* plants. (B–D) PETRA analysis of telomeres on specific chromosome arms in wild type and *parp* mutants. Primer naming convention is the same as in the Figure 6 legend. (B) Analysis of the telomeres on chromosome arms 1L and 2R. DNA was from pooled seedlings. (C) Analysis of four different chromosome arms for individual *parp1*, *parp2,* and *parp1 parp2* mutant plants. Chromosome arms tested are listed below the lanes. (D) Analysis of wild type and G4 *tert* pooled seedlings grown in either 0.6% DMSO or 5 mM 3-AB/0.6% DMSO using the 2R primer. Molecular weight markers are shown to the left of each gel.

To investigate whether PARPs act in concert with telomerase to promote telomere stability, TF-PCR was conducted with 3-AB-treated wild type and G4 *tert* seedlings. A background level of TF-PCR products was observed with one of the wild type seedling samples, while abundant products were evident in two G4 *tert* mutants (Figure 6B). One of the 3-AB-treated G4 *tert* samples displayed telomere fusions, but their abundance was comparable to untreated G4 *tert*. Moreover, two other 3-AB-treated G4 *tert* seedling samples showed no evidence of telomere fusion at all. This result is likely to reflect the stochastic onset of telomere dysfunction among individual *tert* mutant seedlings [80]. End-to-end chromosome fusions are detected when telomeres reach a critical length of about 1 kb [84]. At least a subset of telomeres in these plants are above this threshold (see Figure 7D below). We cannot rule out the possibility that PARPs promote some degree of chromosome end-joining reactions in *Arabidopsis*. Recently, *A. thaliana* PARP1 and PARP2 were shown to be involved in microhomology-mediated end-joining [79], a function similar to human PARPs in the alternative pathway for NHEJ [94]. Although, multiple mechanisms have been implicated in the fusion of dysfunctional telomeres [84], [95], the canonical NHEJ pathway is intact in this setting and thus it is unlikely that PARPs play a significant role in telomere fusion.

### PARPs do not Make a Significant Contribution to Telomere Length Maintenance in *Arabidopsis*

Our negative results for TF-PCR do not preclude the possibility that telomeres were modestly shortened in PARP mutants. To test this possibility, we monitored bulk telomere length in *parp1 and parp2* single mutants and several double mutants using terminal restriction fragment (TRF) analysis. Telomeres in these plants, including the wild type control, were within the typical size range for the Col-0 ecotype of *A. thaliana*, 2–5 kb (Figure 7A). We detected some variability in telomere length between plants doubly deficient for PARP1 and PARP2 (Figure 7A). While four of the double mutants we analyzed had telomere lengths similar to wild type and the *parp1* and *parp2* single mutants, the telomere tracts for one individual double mutant plant were shorter. To obtain another measure of telomere length, we used Primer Extension Telomere Length Amplification (PETRA) to examine the telomere length distribution on four different chromosome arms (Figure 7B and C). As with TRF analysis, PETRA showed that telomeres in single *parp1* or *parp2* mutants did not differ perceptively from wild type (Figure 7B). The same result was obtained for double mutants as well as 3-AB-treated plants (Figure 7C and D). Finally, to determine if PARPs contribute to telomere length maintenance in the absence of TERT, PETRA was conducted on G4 *tert* mutants in the presence or absence of 3-AB. Telomeres in *tert* seedlings treated with 3-AB showed no significant size difference from the corresponding DMSO controls (Figure 7D). Thus, while we cannot rule out a role for PARPs in modulating telomere length in *A. thaliana*, our findings argue that any contribution is likely to be very modest.

## Discussion

Our investigation of *Arabidopsis* PARPs adds to the growing body of research describing the importance of PARPs in multiple cellular processes in eukaryotes. As expected, we verified a role for PARP1 and PARP2 in the plant DNA damage response. Interestingly, although expression of *PARP1* and *PARP2* was induced by genotoxic stress, *PARP3* expression was not. Hence, PARP3 may have functions independent of the DDR. Our results do not eliminate the possibility that PARP3 contributes to the DDR or to DNA repair. Because PARP3 is highly expressed in seeds, it may be critical for the DDR in this setting. When seeds are imbibed with water for germination, reactive oxygen species are produced that can damage DNA, creating an environment where DNA repair is critical to maintain the genome of the developing embryo [96]. Thus, *PARP3* may be important for this DDR in seeds as its expression increases dramatically with seed imbibition [97].

The unknown function of PARP3 leads to the bigger question of whether there is functional overlap between the three *A. thaliana* PARPs that have ADP-ribosylation activity. Our data indicate that PARP expression is controlled by a regulatory network. When *parp1* or *parp2* are absent, the other two PARPs are upregulated. This observation could indicate that PARPs are interchangeable and can compensate for the loss of other PARPs. In this scenario, PARPs could be activated through either the same or parallel pathways. Our finding that combined loss of PARP1 and PARP2 causes increased sensitivity to genotoxic stress is consistent with the latter hypothesis. Alternatively, or possibly in addition, the PARPs may directly regulate expression of each other, perhaps to prevent too much enzymatic activity, which would deplete cellular NAD^+^. There are likely highly complex levels of PARP regulation involved that could vary depending on the needs of the plant. Further experimentation, including the measurement of PARP activity, will be required elucidate the biochemical targets and regulatory pathways of the plant PARPs.

The role of DNA repair proteins in telomere biology is paradoxical. Although one of the main functions of telomeres is to hide chromosome ends from the DNA repair machinery, telomere maintenance requires contributions from numerous repair proteins. This situation raises questions about the evolutionary origins for the telomeric role of DNA damage proteins. While several scenarios can be envisioned, one intriguing possibility is that eukaryotes co-opted the DDR proteins as a way to adapt to the increasing reliance on dynamic telomere regulation in the control of genome stability and cell proliferation.

Our analysis of *Arabidopsis* PARPs uncovered no strong evidence for a role in modulating telomerase enzyme activity, telomere length or chromosome end protection. We detected modest telomere shortening in one of five plants deficient in both PARP1 and PARP2. Why this particular individual had shorter telomeres is unclear. Telomere measurements on four different chromosome arms in the double mutants and in plants treated with 3-AB did not reveal telomere length perturbations, but it is possible that one or more of the telomeres we did not assay falls below the wild type range. It is also possible that telomere-related phenotypes derived from the loss of PARP are not fully penetrant. This hypothesis could be tested by increasing the number of double *parp1 parp2* mutants analyzed. On the other hand, the complete absence of telomere fusions in PARP deficient plants supports the conclusion that telomere length is not significantly altered in these plants. One limitation of using whole plant tissue for telomeric analysis is the possibility that the dysfunction occurs only in a subset of cells and cannot be detected over the normal phenotype which is present in the majority of cells. In our study, we saw defects in root development in 3-AB-treated seedlings. It is possible that the telomeric function of PARPs could be restricted to the root meristem. Our standard assays would not detect such a phenotype. Taken together, our data indicate that any contribution PARPs make to telomere length maintenance in *A. thaliana* is minor or occurs in a small subset of cells.

The large family of PARPs in vertebrates (at least seventeen in humans versus nine in *A. thaliana*) may have afforded them “genetic space” to acquire new telomere-specific functions. Notably, the tankyrases, arguably the most important in human telomere biology, are absent from plant genomes. Thus, telomere-related function of PARPs may have evolved in parallel with mechanisms that regulate telomerase activity and telomere length. PARPs and tankyrases function mainly through interaction with and modification of the shelterin components TRF1 and TRF2. *Arabidopsis* has six putative TRF-like proteins that can bind telomeric dsDNA *in vitro* [7]. Although one of the TRF-like proteins, AtTBP1, negatively regulates telomere length [8], the *in vivo* functions of the others are largely unknown, and appear to be redundant (L. Vespa, Z. Karamysheva, and D. Shippen, unpublished data). It is conceivable that telomere-related functions of *Arabidopsis* PARPs are masked by multiple TRFs, which compensate for each other when one is removed from telomeres due to PARylation.

Finally, plants may not have commandeered PARPs to modulate telomere dynamics, because they do not need to so delicately balance telomere length and telomerase activity the way humans do to avert metastatic cancer. In support of this hypothesis, the model yeasts *Saccharomyces cerevisiae* and *Schizosaccharomyces pombe* completely lack PARP orthologs [28]. Furthermore, mice deficient in Tankyrase1 or Tankyrase2 have normal telomeres over multiple generations [35], [98], [99], and while some investigators report telomere shortening and other telomere aberrations in PARP-deficient mice [56], [91], [93], others have been unable to detect telomere defects [55], [92], [100]. Mice, in contrast to humans, have long telomeres and little repression of telomerase occurs, presumably because their short lifespan does not require a strict mechanism to induce replicative senescence and thereby limit the accumulation of cancer-causing mutations [101]. One attractive hypothesis is that the telomeric function of human PARPs reflects the acquisition of an additional layer of fine-tuning for telomere length regulation.
